# Factors associated with caregiver responsive and non-responsive feeding styles in Clark County, Nevada

**DOI:** 10.1017/S1368980025000096

**Published:** 2025-01-30

**Authors:** Amanda Castelo Saragosa, Sheniz Moonie, Christopher Johansen, Alyssa N Crittenden, Gabriela Buccini

**Affiliations:** 1 School of Public Health, Department of Social and Behavioral Health, University of Nevada, Las Vegas, Las Vegas, NV, USA; 2 School of Public Health, Department of Epidemiology, University of Nevada, Las Vegas, Las Vegas, NV, USA; 3 College of Liberal Arts, Department of Anthropology, Univeristy of Nevada, Las Vegas, Las Vegas, NV, USA; 4 Graduate College, University of Nevada, Las Vegas, Las Vegas, NV, USA

**Keywords:** Early childhood obesity, Responsive feeding, Infant feeding style, Socio-ecological factors, Cross-sectional

## Abstract

**Objective:**

Early childhood obesity (ECO) significantly increased in the USA. ECO interventions lack focus on the prevention of ECO for infants under 2. Caregiver’s feeding styles (CFS) have been shown to affect ECO development, but studies on CFS are limited. This study examined socio-ecological factors associated with CFS for infants under 2 in Nevada.

**Design:**

This cross-sectional study utilising a survey examined the five CFS constructs: responsive (RP), non-responsive (NRP) Laissez-Faire, NRP pressuring, NRP restrictive and NRP indulgent. Descriptive analysis and logistic regression following a hierarchical modelling approach were used to determine the associations between the CFS constructs and socio-ecological factors (e.g. household, maternal mental health and infant feeding).

**Setting:**

Clark County, Nevada.

**Participants:**

304 caregivers with infants under 2.

**Results:**

NRP-feeding styles were associated with low-income households (e.g. NRP restrictive (adjusted OR (AOR) = 2·60, 95 % CI (1·01, 6·71))), water insecurity (e.g. NRP pressuring (AOR = 2·46, 95 % CI (1·00, 6·06)), young mothers (e.g. NRP-Laissez-Faire (AOR = 2·39, 95 % CI (1·00, 5·84))), lower maternal education (e.g. RP (AOR = 0·58, 95 % CI (0·33, 1·00))), mild risk for depression (e.g. NRP restrictive (AOR = 0·50, 95 % CI (0·28, 0·90))) and a moderate to severe risk for anxiety (e.g. NRP pressuring (AOR = 0·32, 95 % CI (0·14, 0·74))). There were no associations between infant-feeding factors and RP feeding.

**Conclusion:**

Our study identified socio-ecological factors associated with dissimilarities in CFS in Nevada. These findings can be used to tailor educational approaches to address disparities in ECO.

Early childhood obesity (ECO) has tripled over the last 40 years, reaching epidemic levels in the USA, with nearly one-third of the US children and adolescents being classified as overweight or obese^(1–6)^. ECO has been shown to cause short- and long-term comorbidities, including hypertension, high cholesterol, diabetes and increased risk for obesity during adulthood^(4–8)^. Moreover, obesity is considered a form of early childhood malnutrition that can coexist with other forms of malnutrition, such as micronutrient deficiencies^(9,10)^. ECO is influenced by numerous components, making it a challenge to address^(11)^. Some factors that affect ECO include a child’s built environment, maternal poor nutritional knowledge, obesity, educational attainment, race/ethnicity, water insecurity and cultural norms about food consumption^(1,3,6)^. Currently, ECO has low rates of resolution and high rates of worsening or relapse after short-term treatments^(7)^. This is an issue because obesity which begins in childhood and prolongs through adulthood becomes more complicated to treat^(5,7)^. Although there have been many advances in ECO research thus far, there is inadequate evidence on how young children develop obesogenic behaviours, particularly in low-socio-economic families^(3)^. Therefore, identifying risk factors for ECO and developing public health prevention strategies to address them is critical to preventing adult obesity and increasing prevalence and obesity-related health risks^(1,5)^. Additionally, studies have found that ECO begins during a child’s first 1000 d (conception to two years) and is a critical period for prevention; however, data on obesity prevention for infants under two are minimal^(12,13)^. Therefore, there is a need to focus on obesity prevention for infants under 2.

One factor that has sparked interest in ECO prevention is caregivers’ feeding styles (CFS), how caregivers maintain or modify their child’s eating behaviours and feeding environment^(2,4,14)^. There are two feeding styles: responsive (RP) and non-responsive (NRP). An RP feeding style is when a parent is attentive to the child’s hunger and satiety cues and monitors the quality of the child’s diet^(2)^. An NRP feeding style is the opposite of RP feeding, where parents engage in negative feeding behaviours with their children^(14)^. For example, a caregiver who exhibits an NRP-feeding style could control their child’s diet quality or quantity and use food as a soother^(2)^. RP feeding has been shown to create healthy eating habits and growth and reduce child under- and overnutrition, while NRP feeding has been shown to create overnutrition or obesity^(14)^. Therefore, assessing factors associated with dissimilarities among CFS may yield information on the causes of ECO^(15)^ and could provide public health professionals with new insights into the prevention mechanisms of ECO^(2)^. Prior studies have exhibited factors associated with CFS, including caregiver time constraints^(14)^, child’s weight status^(14,16)^, caregiver weight status^(14)^, income^(14,16)^, caregiver beliefs and perceptions^(14)^, race and ethnicity^(14,16,17)^, caregiver self-efficacy^(18)^, social support^(19)^, education and knowledge^(16,19,20)^, depression^(16)^, household food insecurity^(20)^ and breastfeeding^(20)^. However, although many of these factors have already been studied, most were conducted in other countries and not in the USA and may not be generalisable to the US population^(14,16,18–20)^. Therefore, this study will analyse similar factors to see how they are associated with CFS in one large urban geographical area in the USA.

The socio-ecological model (sem) is a theoretical framework that helps researchers understand the factors influencing health and behaviours^(21)^. The sem focuses on how it is essential to consider factors beyond an individual’s immediate context to understand their health and behaviours^(21)^. The sem has five levels: intrapersonal (knowledge, behaviours, beliefs and attitudes), interpersonal (families, friends, social support), institutional (workplaces, schools and organisations), community (cities, neighbourhoods, resources) and policy (federal, state and local legislation)^(21)^. This study will utilise the sem to guide the assessment of the different socio-ecological factors associated with CFS. To our knowledge, no other studies have used the socio-ecological framework to organise and assess the actors influencing CFS. Thus, this study aimed to analyse the socio-ecological factors associated with caregivers’ RP and NRP-feeding styles.

## Methods

### Study design

This cross-sectional study utilised a survey to examine the socio-ecological characteristics of mother–infant dyads on CFS. CFS was classified into five constructs: RP feeding, NRP Laissez-Faire feeding, NRP-pressuring feeding, NRP-indulgent feeding and NRP-restrictive feeding. The study’s protocol was approved by the University of Nevada, Las Vegas’s Institutional Review Board (Protocol UNLV-2022-372). Participation in this study was voluntary. No personal information was collected, informed consent was obtained at the beginning of each survey and answers were kept completely anonymous. The Strengthening the Reporting of Observational Studies in Epidemiology (STROBE) Statement was used to guide the reporting of this study (Appendix A).

### Study setting

The study was conducted in Clark County, which accounts for 73 % of Nevada’s population^(22)^. Per the 2020 State of Nevada Annual Obesity Report, 11·1 % of children entering kindergarten were overweight, and 21·3 % of those children were obese^(23)^. Additionally, 11·6 % of participants in the Special Supplemental Nutrition Program for Women, Infants and Children (WIC) who were between 2 and 4 years old in Nevada are considered obese^(24)^.

### Participants

Inclusion criteria included any mother/caregiver who was 18 and older, who had an infant ages 0–23 months (birth to under 2 years old) and who resided in Clark County. This study excluded any infant with special needs that prevented them from adopting optimal feeding practices, including infants with specific illnesses/needs (Down syndrome, cleft lip or palate, congenital heart disease, neurological conditions or cardiac problems).

### Sampling

This study utilised a snowball sampling approach where key stakeholders of the study setting were identified and subsequently asked to share the study with others they know^(25)^. This study recruited mothers from Baby-friendly hospitals, birth centres, paediatric centres, lactation centres and WIC centres within Clark County. Additionally, surveys were dispersed through social media platforms (Facebook and Instagram). With the assistance of a statistician, two sample sizes were calculated: the sample needed for the survey and the sample to test our hypothesis that looks at the socio-ecological factors associated with CFS. The survey sample size was determined using live births in Clark County. According to Southern Nevada Health District Vital Records Statistics, there were 25 493 live births in 2021^(26)^. Using a 95 % CI, a 5 % margin of error, and assuming there will be a completion of 50 %, we determined a sample size of 379 mother/caregiver–infant dyads. The minimum sample size required to test the study hypothesis was estimated using G*power version 9·0·1. Results from the power analysis indicated that the minimum required sample size to achieve 80 % power with a moderate effect size (Cohen’s d = 0·5), at a significance criterion α = .05, was *n* 71 for each feeding style for logistic regression. Thus, the analytical sample consisted of 304 mothers in Clark County with children between 0 and 23 months old, which was deemed sufficient.

### Survey development

**Fig. 1:**
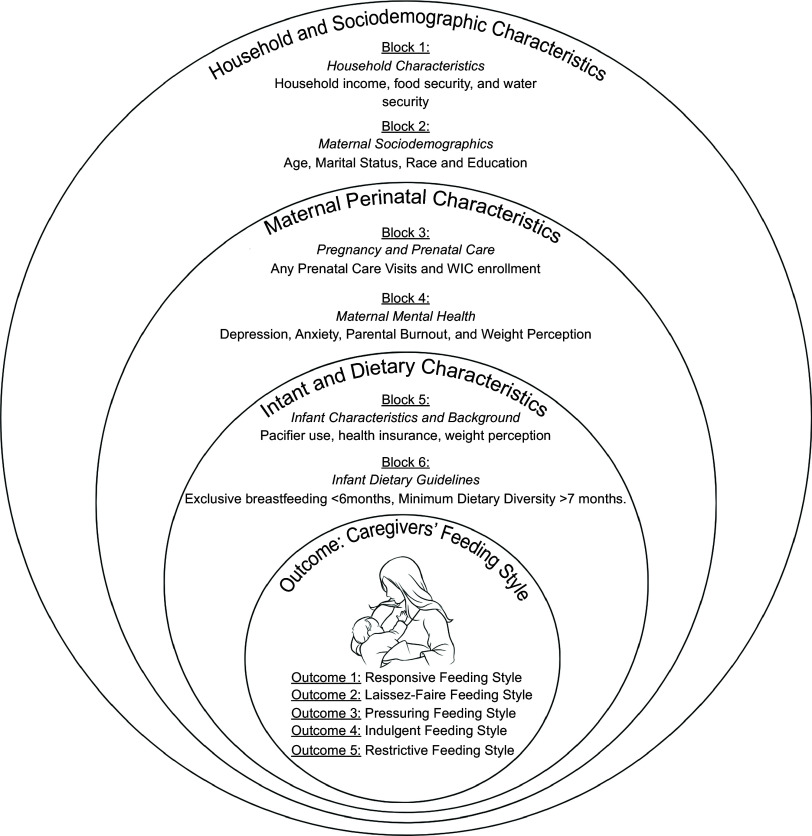
Socio-ecological model depicting the different socio-ecological levels that influence caregiver’s feeding styles.

The 2022 Early Responsive Nurturing Care (EARN) survey sections included household and socio-demographic characteristics, maternal perinatal characteristics, infant and dietary characteristics and caregiver feeding styles as outlined in Fig. 1. It included questions from validated instruments detailed in the measurement section. The survey was developed in English and translated into Spanish; both versions were available to participants.

### Measurement

#### Outcome: caregiver feeding styles

The outcome of this study was CFS collected using the Infant Feeding Style Questionnaire (Fig. 1). Outcome data including the five different CFS were collected utilising the Infant Feeding Style Questionnaire^(2)^, which is a self-report instrument that measures feeding beliefs and behaviours among caregivers of infants and young children^(2)^. Although the Infant Feeding Style Questionnaire includes a substantial number of questions (*n* 83) (Appendix B), it was chosen for this study for many reasons. This questionnaire is very well organised and categorises the questions into different feeding styles (Laissez-Faire, pressuring, restrictive, RP, indulgence)^(2)^. The Infant Feeding Style Questionnaire is a valid and reliable instrument for the US population, has been used on infants ages 3–24 months and includes all RP feeding measures compared to other valid instruments^(4)^. The overall mean scores from each feeding style were calculated to classify whether the participants exhibited the five different feeding styles. If a participant scored above the mean with respect to a specific feeding style, they were classified as exhibiting that style. They were classified as not exhibiting that feeding style if they scored below the mean. The outcome can be classified into five constructs of feeding styles: RP, Laissez-Faire, pressuring, indulgent and restrictive (Appendix C).

An RP feeding style is when a caregiver monitors their child’s diet quality and is attentive to their hunger and satiety cues^(2)^. For data analysis, the reference category was considered the negative response to RP; thus, the analysis shows the odds ratios to be a NRP feeder (*v*. an RP feeder). An NRP Laissez-Faire feeding style is when a caregiver does not restrict their child’s diet quantity or quality and minimally interacts during feeding^(2)^. An NRP-pressuring feeding style is when a caregiver force-feeds their child because they worry about the amount of food they are consuming while also using food as a soother^(2)^. An NRP-indulgent feeding style is when a caregiver sets no restrictions on the quality and quantity of the child’s food^(2)^. Lastly, an NRP-restrictive feeding style is when a caregiver limits the amount and type of food their child consumes^(2)^. For data analysis, the reference category was considered the positive response to the NRP.

#### Covariates

We will be utilising the sem as outlined in Fig. 1 to guide the assessment of the socio-ecological factors. This study’s covariates were selected using the conceptual hierarchical framework and evidence from previous studies that connect the covariates and the outcomes (classifications found in Appendix D)^(14,18,19,27–29)^. Variables were categorised based on their associations with other variables and the study outcomes. There were three levels of covariates, including household and socio-demographic characteristics (household characteristics and maternal socio-demographics), maternal perinatal characteristics (pregnancy and prenatal care and maternal mental health), and infant and dietary characteristics (infant characteristics, background and dietary guidelines) as depicted in Fig. 1.

#### Household and socio-demographic characteristics

##### Block 1: household characteristics

Household characteristic data was collected using questions related to (a) household income, (b) food security and (c) water security.

- To measure household income, participants were asked to self-report their household income picking from different ranges of income values.
- To measure food security, the Hunger Vital Sign (HVS)^(30)^ was used. The HVS is a two-item screening tool to measure risk for household food insecurity based on the US Household Food Security Survey Model^(30)^. It is a validated tool for children and adults and was chosen because it is a simple form to identify food insecurity risk^(30)^. Individuals answered the questions from ‘never true’, ‘sometimes true’ or ‘often true’^(30)^. If they answered ‘sometimes true’ or ‘often true’ to either of the questions, they were considered at risk for food insecurity^(30)^.
- To measure water security, the Household Water Insecurity Access Survey (HWIAS)^(29)^ was used. HWIAS is an eight-item self-reported questionnaire that measures household water insecurity and was developed based on the household food insecurity access scale^(31)^. This questionnaire is a valid and reliable instrument in developing countries^(31)^. Here, we used only the most severe question from the instrument to capture the presence/absence of water insecurity. We did so to be considerate of the length of the final survey to mitigate the potential research fatigue of participants. The question used was ‘Within the past 12 months, we worried about not having enough money to afford access to clean water (i.e. drinking water, bathing/washing hands, washing clothes or any other needs)’, and individuals could answer ‘never true’, ‘sometimes true’ or ‘often true’^(31)^. If they answered either sometimes or often true, they were classified as at risk for water insecurity^(31)^.

##### Block 2: maternal socio-demographics

Maternal socio-demographic data were collected using questions related to (a) maternal age, (b) marital status, (c) maternal race and (d) maternal education.

- Maternal age was measured by asking the participants to self-report the mothers’ age selecting from a different set of age ranges.
- Marital status was measured by asking the participants to self-report whether they were living with or without a partner.
- Maternal race was measured by asking the participants to self-report their race by selecting from a set of race categories. They were also then asked to self-report whether they were from Latina, Hispanic or Spanish origin. From this, they were separated into non-Hispanic white or Hispanic white.
- Maternal education was measured by asking the participants to self-report the higher level of education the mother obtained from a set of education categories.

#### Maternal perinatal characteristics

##### Block 3: pregnancy and prenatal care

Pregnancy and prenatal care data were collected using questions related to (a) prenatal care visits and (b) WIC enrolment.

- Prenatal care was measured by asking the participants to self-report whether they visited a primary care doctor or OB/GYN for prenatal care.
- WIC enrolment was measured by asking participants to self-report whether they were enrolled in the WIC programme.

##### Block 4: maternal mental health

Maternal mental health data were collected using questions related to (a) depression, (b) anxiety, (c) parental burnout and (d) maternal weight perception.

- Maternal depression risk was measured using the Edinburgh Postnatal Depression Scale^(32)^. The Edinburgh Postnatal Depression Scale is a ten-item self-reported instrument determining postpartum depression risk in mothers^(32)^. It was chosen because it is the most commonly used screening questionnaire for identifying risk for postpartum depression, validated and translated into different languages, specifically Spanish^(32)^. Although the Edinburgh Postnatal Depression Scale is normally reserved for mothers with infants between 0 and 12 months, it has been validated to be used on mothers with older children^(33)^. Prior studies have also used this tool to measure mothers with children up to 24-month postpartum depression risk^(34)^. The instrument has a mother report how she has felt during the previous 7 days^(35)^. Responses are scored 0, 1, 2 and 3 based on the seriousness of the symptoms^(35)^. Scoring is as follows: 0–6 ‘risk for no or minimal depression’, 7–13 ‘mild depression,’ 14–19 ‘moderate depression’ and 19–30 ‘severe depression’^(35)^.
- Maternal anxiety risk was measured using the Generalised Anxiety Disorder Assessment^(36)^. The Generalised Anxiety Disorder Assessment-7 is a 7-item self-reported instrument determining general anxiety disorder risk^(36)^. This instrument was chosen because it has been proven valid and reliable across many cultures and is available in different languages, including Spanish^(36)^. The survey asks an individual the severity of their symptoms over the last 2 weeks, from ‘not at all,’ ‘several days’, ‘more than half the days’ and ‘nearly every day’^(37)^. Responses are scored 0, 1, 2 and 3 based on the seriousness of the symptom^(37)^. Scoring is as follows: 0–4 ‘risk for minimal anxiety’, 5–9 ‘mild anxiety’, 10–14 ‘moderate anxiety’ and 15–21 ‘severe anxiety’^(37)^.
- Maternal burnout risk was measured using the Brief Parental Burnout Scale^(38)^. The Brief Parental Burnout Scale is a five-item screening tool to measure an individual’s emotional distress, exhaustion and feelings from being a parent^(38)^. It was chosen because it is a validated and short tool based on the Parental Burnout Assessment^(38)^. The parents rate their symptoms from A ‘daily’, B ‘once or twice a week’ or C ‘more seldom/never’^(38)^. If a parent answers ‘A’ to at least one question or ‘B’ to at least two questions, they are at risk for parental burnout^(38)^.
- Maternal weight perception was measured by asking the mothers to self-report how they would describe their weight based on a set of weight categories.

#### Infant and dietary characteristics

##### Block 5: infant characteristics and background

Infant characteristics and background data were collected using questions related to (a) pacifier use, (b) infant health insurance and (c) perception of infants’ weight.

- Infant age was measured by asking participants to indicate their last child’ s age selecting from a different set of age ranges.
- Pacifier use was measured by asking the participants if their infant used a pacifier in the last 24 h.
- Infant health insurance was measured by asking participants to indicate what type of medical insurance their infant has from a set of insurance categories.
- Perception of infants’ weight was measured by asking participants to self-report how they would describe their infants weight based on a set of weight categories.

##### Block 6: infant dietary guidelines

A self-reported questionnaire was developed based on the World Health Organization’s recommendations on how to assess infant and young child feeding^(39)^. Caregivers were asked to select all the food groups that the child consumed in the last 24 h, including breast milk, grains/roots/tubers/plantains, pulses/nuts/seeds, dairy products, flesh foods, eggs, vitamin A-rich fruits/vegetables and other fruits/vegetables^(39)^. This questionnaire was used to identify whether a caregiver was reaching the infants dietary guidelines, including exclusive breastfeeding <6 months (i.e. an infant was fed with only breastmilk, with no other types of food or drinks or water^(39)^) and minimum dietary diversity >7 months (i.e. a child (breastfed or non-breastfed) was fed at least five out of eight food groups: (1) breast milk, (2) grains/roots/tubers/plantains, (3) pulses/nuts/seeds, (4) dairy products, (5) flesh foods, (6) eggs, (7) vitamin A rich fruits/vegetables and (8) other fruits/vegetables^(39)^). For analysis, meeting the infant dietary guidelines was classified as ‘yes’ when an infant <6 months reported exclusive breastfeeding or an infant >7 months reported minimum dietary diversity.

### Data analysis

The survey data were collected via Qualtrics and exported to STATA se 17 for analysis. First, descriptive analysis was performed for the outcomes and covariates, including the mean, sd and frequency distribution. Second, bivariate correlations were performed to determine the associations between the outcome and covariates. Covariates were included in a multivariate model when they had an association with a *P*-value <0·20 in the bivariate analysis. A collinearity test was performed, and no collinearity violations were detected. To identify the associations of CFS and covariates a logistic regression following a hierarchical modelling approach with robust variance was performed to generate the adjusted OR (AOR) and corresponding 95 % CI.

Each feeding style was analysed separately. For each feeding style, the following approach was followed: Model 1 of the analysis included variables from block 1 (household characteristics and infant age) and remained the control for the forthcoming models. Model 2 of the analysis included variables from block 2 (maternal socio-demographics) was adjusted by including model 1 and remained the control for the subsequent models. Model 3 of the analysis included variables from block 3 (pregnancy and prenatal care) and was adjusted by including models 1 and 2, remaining as the control for the following models. Model 4 of the analysis included variables from block 4 (maternal mental health), was adjusted by including the three previous models and remained the control for the subsequent models. Model 5 of the analysis included variables from block 5 (infant characteristics), was adjusted by including the previous four models and remained the control for the subsequent models. Lastly, model 6 of the analysis included variables from block 6 (infant feeding) and was adjusted by including variables in the previous five models. A *P*-value of <0·05 was the criterion for statistical significance at each level to evaluate the association between the covariates and the outcome. All covariates included in the hierarchical modelling approach were maintained in all model levels regardless of the significance attenuating, as these data provide important adjustments to the parameter estimates in the final models.

## Results

### Descriptive analysis

A total of 304 mothers in Clark County with infants between 0 and 23 months old responded to the survey. Mothers (*n* 304) could be classified into one or more feeding styles. Of those who answered the questions for each feeding style, 53 % were classified as RP feeders (*n* 153), 47 % were classified as NRP-Laissez-Faire feeders (*n* 138), 43 % were classified as NRP-pressuring feeders (*n* 126), 36 % were classified as NRP-indulgent feeders (*n* 93) and 50 % of them were classified as NRP-restrictive feeders (*n* 149) (Table 1). The majority of the mothers were between the ages 24 and 35 years (*n* 196, 65 %), middle income (*n* 201, 66 %), lived with their partners (*n* 282, 93 %), Hispanic (*n* 156, 51 %) and had a secondary or college level education (*n* 222, 73 %). Approximately 30 % of the respondents were at risk for food insecurity (*n* 92), and 13 % were at risk for water security (*n* 39). Most of the mothers were not enrolled in WIC (*n* 244; 80 %), and almost all had some type of prenatal care (*n* 285; 94 %). Approximately 23 % were at risk for moderate to severe depression (*n* 67), 23 % were at risk for moderate to severe anxiety (*n* 70) and 81 % were at risk for parental burnout (*n* 245). Additionally, the majority of the mothers believed they were overweight (*n* 210, 66·1 %). Among infants, most were between the ages of 12–23 months (*n* 131, 43 %), did not use pacifiers (*n* 175, 57·6 %), had non-government-provided insurance (*n* 223, 73 %), were perceived by their mother as having normal weight (*n* 251, 82·5 %) and were adequately fed (*n* 180, 64·3 %) (Table 1).

**Table 1.** Descriptive analysis of feeding styles, household characteristics, maternal socio-demographics, prenatal care, maternal mental health, infant characteristics and infant feeding, 2023

Study variables full sample (*n* 304)	%	n*
**Caregiver feeding style(s)**		
Responsive (RP) (*n* 287)		
Yes	53·3	153
No	46·7	134
Non-responsive Laissez-Faire (NRP-LF) (*n* 293)		
Yes	47·1	138
No	52·9	155
Non-responsive pressuring (NRP-PR) (*n* 293)		
Yes	43·0	126
No	57·0	167
Non-responsive indulgence (NRP-ID) (*n* 261)		
Yes	35·6	93
No	64·4	168
Non-responsive restrictive (NRP-RS) (*n* 296)		
Yes	50·34	149
No	49·66	147
**Block 1**		
Infants age (constant)		
Under 6 months	33·9	103
Between 7 and 11 months	23·0	70
Between 12 and 23 months	43·1	131
Household income		
Low income (less than $49 999)	21·7	66
Middle income ($50 000–$149 999)	66·1	201
Upper income (more than $150 000)	12·2	37
Food security		
Food secure	69·7	212
Food insecure	30·3	92
Water security		
Water secure	87·2	265
Water insecure	12·8	39
**Block 2**		
Mother’s age		
18–24	12·5	38
25–34	64·5	196
35–44	23·0	70
Marital status		
Living without a partner (single, widowed, separated)	7·2	22
Living with a partner (married, living together)	92·8	282
Non-Hispanic white		
Yes	48·7	148
No	51·3	156
Mother’s education		
Secondary or college	73·0	222
Graduate	26·9	82
**Block 3**		
Any prenatal care (PNC) visits		
Yes	93·8	285
No	6·3	19
WIC enrolment		
Yes	19·7	60
No	80·3	244
**Block 4**		
Mother’s risk for depression (*n* 292)		
No/minimal	41·1	120
Mild	35·9	105
Moderate/severe	22·9	67
Mother’s risk for anxiety (*n* 297)		
No/minimal	34·7	103
Mild	41·8	124
Moderate/severe	23·6	70
Mother’s risk for burnout (*n* 303)		
Burnout risk	80·9	245
No burnout	19·1	58
Mother’s weight perception		
Underweight	2·3	7
Normal weight	31·6	96
Overweight	66·1	201
**Block 5**		
Pacifier use		
Yes	42·5	129
No	57·6	175
Infants insurance (*n* 303)		
Government	26·4	80
Non-government	73·6	223
Perception of infant’s weight		
Underweight	9·2	28
Normal weight	82·6	251
Overweight	8·2	25
**Block 6**		
Infant dietary guidelines (exclusive <6 months and complementary <6 months) (*n* 280)		
Yes	64·3	180
No	35·7	100

WIC, Women, Infants and Children.

### Bivariate analysis

NRP feeding was more frequent among mothers between the ages of 25–34 (*n* 93, 51 %), who identified as Hispanic (*n* 74, 51 %), and had a graduate degree (*n* 43, 56 %) compared with their comparison groups. NRP Laissez-Faire feeding was more frequent among mothers who classified as living in low-income households (*n* 37, 58 %), were at risk for food insecurity (*n* 50, 56 %), and between the ages 18–24 (*n* 25, 68 %), were enrolled in WIC (*n* 34, 57 %), perceived themselves as overweight (*n* 100, 52 %), whose infant used pacifiers (*n* 69, 55 %) and had government insurance (*n* 47, 60 %) compared to their comparison groups. NRP-pressuring feeding was more frequent among mothers who were classified as living in a low-income household (*n* 42, 67 %), were at risk for food (*n* 45, 52 %) and water (*n* 25, 66 %) insecurity, were living without a partner (*n* 12, 59 %), were Hispanic (*n* 76, 51 %), were enrolled in WIC (*n* 29, 52 %) and whose infant has government insurance (*n* 40, 54 %) and perceived their infant as underweight (*n* 15, 53 %) or overweight (*n* 16, 64 %) compared to their comparison groups. NRP-indulgent feeding style was more frequent among mothers aged 18–24 (*n* 18, 56 %) than their comparison groups. Lastly, NRP-restrictive feeding style was more frequent among mothers who classified living in a low-income household (*n* 44, 69 %), were at risk for food (*n* 53, 59 %) and water insecurity (*n* 25, 64 %) insecurity, between the ages of 18–24 (*n* 25, 66 %) or 35–44 (*n* 39, 58 %), were Hispanic (*n* 86, 57 %), enrolled in WIC (*n* 39, 66 %) and had no to minimal risk (*n* 65, 56 %) or moderate to severe risk (*n* 34, 51·52 %) for depression, if their infant had government insurance (*n* 44, 56 %) and if they perceived their infant as overweight (*n* 16, 64 %) compared to their comparison groups (Table 2).

**Table 2.** Bivariate analysis of feeding styles by household characteristics, maternal socio-demographics, prenatal care, maternal mental health, infant characteristics and infant feeding, 2023

	Responsive feeding			Non-responsive feeding											
Variables	Non-responsive style (*n* 287)		*P*-value	Non-responsive Laissez-Faire style (*n* 293)		*P*-value	Non-responsive pressuring style (*n* 293)		*P*-value	Non-responsive indulgence style (*n* 261)		*P*-value	Non-responsive restrictive style (*n* 296)		*P*-value
	*n*	%		*n*	%		*n*	%		*n*	%		*n*	%	
**Block 1**															
Household income															
Upper income	17	47·22	0·977	12	32·43	**0·045** ^*^	11	29·73	**0·000** ^*^	8	8·60	0·457	16	45·71	**0·004** ^*^
Middle income	86	46·24		89	46·35		73	37·82		64	35·75		89	45·18	
Low income	31	47·69		37	57·81		42	66·67		21	40·38		44	68·75	
Food security															
Food secure	87	44·16	0·204	88	43·35	**0·053** ^*^	81	39·32	**0·050** ^*^	61	33·70	0·327	96	46·60	**0·052** ^*^
Food insecure	47	52·22		50	55·56		45	51·72		32	40·00		53	58·89	
Water security															
Water Secure	116	46·59	0·928	117	45·70	0·208	101	39·61	**0·002** ^*^	78	34·36	0·268	124	48·25	**0·065** ^*^
Water Insecure	18	47·37		21	56·76		25	65·79		15	44·12		25	64·10	
**Block 2**															
Mother’s age															
35–44	28	41·79	**0·108** ^*^	27	38·57	**0·016** ^*^	27	39·13	0·245	17	27·42	**0·020** ^*^	39	58·21	**0·019** ^*^
25–34	93	51·10		86	46·24		79	42·02		58	34·73		85	44·50	
18–24	13	34·21		25	67·57		20	55·56		18	56·25		25	65·79	
Marital status															
Living with a partner	124	46·79	0·904	128	47·23	0·872	113	41·70	**0·113** ^*^	89	37·08	**0·098** ^*^	137	50·00	0·682
Living without a partner	10	45·45		10	45·45		13	59·09		4	19·05		12	54·55	
Non-Hispanic White															
Yes	60	42·55	**0·167** ^*^	61	43·26	0·205	50	34·38	**0·004** ^*^	42	32·81	0·351	63	43·45	**0·020** ^*^
No	74	50·68		77	50·66		76	51·35		51	38·35		86	56·95	
Mother’s education															
Graduate degree	43	55·84	**0·060** ^*^	31	39·24	**0·102** ^*^	27	33·33	**0·039** ^*^	26	36·62	0·839	36	45·00	0·264
Secondary level or college	91	43·33		107	50·00		99	46·70		67	35·26		113	52·31	
**Block 3**															
Any prenatal care (PNC) visits															
Yes	126	47·01	0·678	132	48·00	0·227	125	45·29	**0·001** ^*^	91	37·60	**0·018** ^*^	39	50·18	0·836
No	8	42·11		6	33·33		1	5·88		2	10·53		10	52·63	
WIC enrolment															
Yes	29	49·15	0·671	34	56·67	**0·096** ^*^	29	51·79	**0·140** ^*^	21	40·38	0·424	39	66·10	**0·007** ^*^
No	105	46·05		104	44·64		97	40·93		72	34·45		110	46·41	
**Block 4**															
Mother’s risk for depression (*n* 276)															
No/Minimal	56	48·70	0·557	56	47·86	0·930	56	48·28	0·267	33	32·04	0·640	65	55·56	**0·098** ^*^
Mild	42	42·86		48	46·15		39	38·61		34	37·78		42	41·18	
Moderate/severe	32	50·79		27	45·00		25	38·46		22	37·93		34	51·52	
Mother’s risk for anxiety (*n* 282) tab															
No/Minimal	47	47·00	0·870	47	46·53	0·982	47	46·53	**0·147** ^*^	29	31·87	**0·191** ^*^	57	55·58	0·332
Mild	52	45·22		56	46·28		55	46·22		36	33·96		59	48·76	
Moderate/severe	33	49·25		31	47·69		22	32·84		27	45·76		30	44·78	
Mother’s risk for burnout															
Burnout risk	109	46·98	0·838	111	46·84	0·853	103	43·10	0·968	79	37·62	0·202	120	50·42	0·951
No burnout	25	45·45		27	48·21		23	43·40		14	28·00		29	50·88	
Mother’s weight perception															
Underweight	2	28·57	0·423	2	28·57	**0·070** ^*^	3	42·86	0·827	3	42·86	0·532	3	42·86	0·916
Normal weight	39	43·33		36	38·71		38	40·43		26	30·95		47	50·00	
Overweight	93	48·95		100	51·81		85	44·27		64	37·65		99	50·77	
**Block 5**															
pacifier use															
Yes	57	47·90	0·730	69	54·76	**0·022** ^*^	58	46·77	0·264	39	36·45	0·818	61	48·41	0·568
No	77	45·83		69	41·32		68	40·24		54	35·06		88	51·76	
Infants insurance (*n* 286)															
Government	33	42·31	0·384	47	60·26	**0·007** ^*^	40	54·05	**0·024** ^*^	30	46·15	**0·044** ^*^	44	56·41	**0·199** ^*^
Non-Government	100	48·08		91	42·52		85	38·99		63	32·31		104	47·93	
Perception of infants weight															
Underweight	8	29·63	**0·169** ^*^	13	46·43	0·641	15	53·57	**0·031** ^*^	9	34·62	0·911	11	39·29	**0·198** ^*^
Normal weight	115	48·63		112	46·28		95	39·58		76	35·35		122	50·21	
Overweight	11	45·83		13	56·52		16	64·00		8	40·00		16	64·00	
**Block 6**															
Infant dietary guidelines (exclusive breastfeeding (<6 months) and complementary feeding (>7 months))															
Yes	80	45·98	0·628	79	45·40	0·421	62	35·43	**0·003** ^*^	52	33·55	0·453	88	49·72	0·834
No	39	42·86		48	50·53		52	54·17		33	38·37		49	51·04	

WIC, Women, Infants and Children.

* *P* < 0.20.

### Multivariate logistic regression analysis

**Fig. 2:**
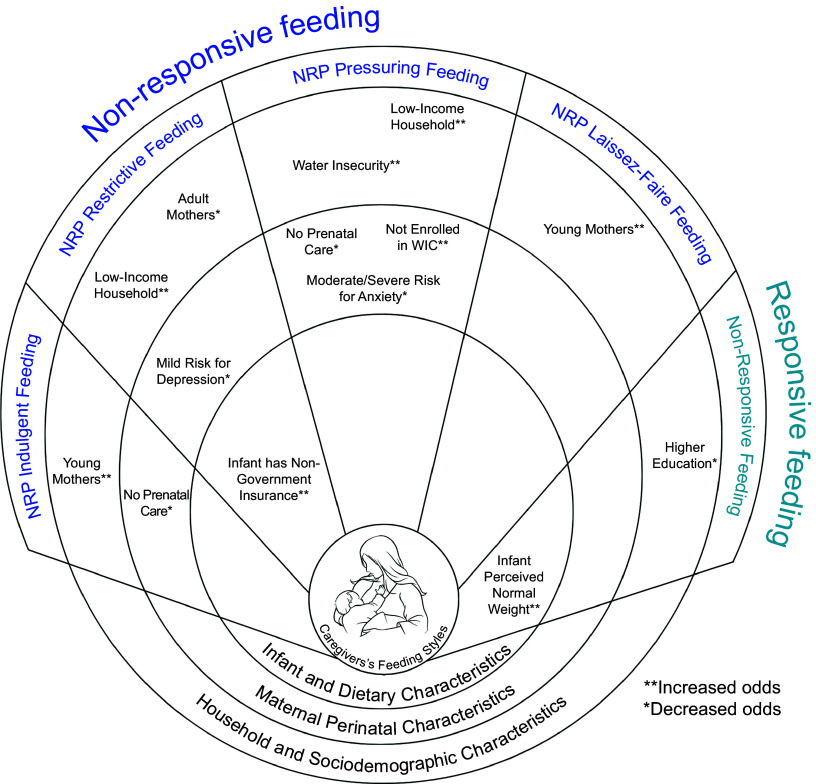
Socio-ecological model depicting the different socio-ecological factors associated with caregivers’ feeding styles organised by socio-ecological levels.

Figure 2 organises the multivariate logistic regression across sem. In model 1, living in a low-income household was associated with NRP-pressuring (AOR = 4·16, 95 % CI (1·54, 11·6)) and NRP-restrictive (AOR = 2·60 (1·01, 6·71)) feeding styles, and having risk for water insecurity was associated with NRP-pressuring feeding style (AOR = 2·46 (1·00, 6·06)). In model 2, mothers aged 18–24 were associated with NRP-Laissez-Faire (AOR = 2·39, 95 % CI (1·00, 5·84)) and NRP-indulgent (AOR = 3·66, 95 % CI (1·45, 9·25)) feeding styles, and mother’s aged 25–34 were associated with an NRP-restrictive (AOR = 0·54, 95 % CI (0·29, 0·98)) feeding style. Also, mothers with a secondary or college education were associated with NRP feeding (AOR = 0·58, 95 % CI (0·33, 1·00)) in model 2. In model 3, a mother having no prenatal care was associated with NRP pressuring (AOR = 0·06, 95 % CI (0·01, 0·52)) and NRP-indulgent (AOR = 0·21, 95 % CI (0·04, 1·00)) feeding styles, and not being enrolled in WIC was associated with a NRP-pressuring feeding style (AOR = 2·47, 95 % CI (1·00, 6·15)). In model 4, the NRP-restrictive feeding style was associated with mothers at risk for mild depression (AOR = 0·50, 95 % CI (0·28, 0·90)), and NRP-pressuring feeding style was associated with mothers at risk for moderate to severe risk for anxiety (AOR = 0·32, 95 % CI (0·14, 0·74)). Lastly, in model 5, NRP-restrictive feeding style was associated with an infant who had non-government insurance (AOR = 2·78, 95 % CI (1·13, 6·82)), and NRP feeding was associated with an infant who was perceived as normal weight (AOR = 2·49, 95 % CI (1·02, 6·06)) (Table 3).

**Table 3:** Logistic regression following a hierarchical modelling approach of feeding styles by household characteristics, maternal socio-demographics, prenatal care, maternal mental health, infant characteristics and infant feeding, adjusted for infant age, 2023

	Responsive Feeding		Non-Responsive Feeding							
Variables	Non-Responsive Style		Non-Responsive Laissez-faire Style		Non-Responsive Pressuring Style		Non-Responsive Indulgence Style		Non-Responsive Restrictive Style	
	AOR	95 % CI	AOR	95 % CI	AOR	95 % CI	AOR	95 % CI	AOR	95 % CI
**Model 1**										
Household income										
Upper income	–		1		1		–		1	
Middle income	–		1·55	0·75, 3·32	1·34	0·61, 2·95	–		1·00	0·47, 2·10
Low income	–		2·11	0·83, 5·36	^*^ **4·16** 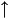	**1·54, 11·6** 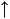	–		^*^ **2·60** 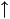	**1·01, 6·71** 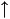
Food security										
Food secure	–		1		1		–		1	
Food insecure	–		1·44	0·81, 2·55	0·73	0·35, 1·49	–		0·96	0·48, 1·89
Water security										
Water secure	–		–		1		–		1	
Water insecure	–		–		^*^ **2·46** 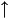	**1·00, 6·06** 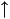	–		1·35	0·55, 3·33
**Model 2**										
Mother’s age										
35–44	1		1		–		1		1	
25–34	1·53	0·85, 2·76	1·23	0·69, 2·19	–		1·36	0·70, 2·64	^*^ **0·54** 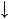	**0·29, 0·98** 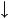
18–24	0·76	0·33, 1·74	^*^ **2·39** 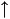	**1·00, 5·84** 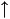	–		^*^ **3·66** 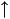	**1·45, 9·24** 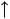	0·95	0·39, 2·35
Marital status										
Living with a partner	–		–		1		1		–	
Living without a partner	–		–		1·40	0·45, 3·64	0·34	0·10, 1·16	–	
Non-Hispanic White										
Yes	1		–		1		–		1	
No	1·57	0·97, 2·55	–		1·54	0·93, 2·57	–		1·40	0·84, 2·31
Mother’s education										
Graduate Degree	1		1		1		–		–	
Secondary level or college	^*^ **0·58** 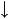	**0·33, 1·00** 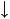	1·16	0·65, 2·07	1·20	0·67, 2·15	–		–	
**Model 3**										
Any prenatal care (PNC) visits										
Yes	–		–		1		1		–	
No	–		–		^*^ **0·06** 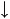	**0·01, 0·52** 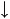	^*^ **0·21** 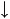	**0·04, 1·00** 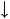	–	
WIC enrolment										
Yes	–		1		1		–		1	
No	–		0·98	0·46, 2·08	^*^ **2·47** 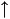	**1·00, 6·15** 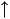	–		0·78	0·35, 1·75
**Model 4**										
Mother’s risk for depression (*n* 276)										
No/Minimal	–		–		–		–		1	
Mild	–		–		–		–		^*^ **0·50** 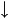	**0·28, 0·90** 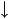
Moderate/Severe	–		–		–		–		0·72	0·36, 1·44
Mother’s risk for anxiety (*n* 282)										
No/Minimal	–		–		1		1		–	
Mild	–		–		0·78	0·43, 1·41	0·91	0·48, 1·71	–	
Moderate/severe	–		–		^*^ **0·32** 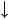	**0·14, 0·74** 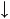	1·72	0·85, 3·47	–	
Mother’s risk for burnout										
Burnout risk	–		–		–		–		–	
No burnout	–		–		–		–		–	
Mother’s weight perception										
Underweight	–		1		–		–		–	
Normal weight	–		1·54	0·25, 9·20	–		–		–	
Overweight	–		2·47	0·42, 14·42	–		–		–	
**Model 5**										
Pacifier use										
Yes	–		1		–		–		–	
No	–		0·66	0·39, 1·09	–		–		–	
Infants insurance (*n* 286)										
Government	–		1		1		1		1	
Non-Government	–		0·52	0·23, 1·16	1·01	0·34, 3·05	0·60	0·30, 1·18	^*^ **2·78** 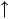	**1·13, 6·82** 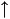
Perception of infants” weight										
Underweight	1		–		1		–		1	
Normal weight	^*^ **2·49** 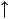	**1·02, 6·06** 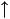	–		0·47	0·19, 1·15	–		2·08	0·84, 5·12
Overweight	2·59	0·77, 8·69	–		1·28	0·35, 4·56	–		2·92	0·89, 9·55
**Model 6**										
Infant dietary guidelines (exclusive breastfeeding (<6 months) and complementary feeding (>6 months))										
Yes	–		–		1		–		–	
No	–		–		1·74	0·90, 3·37	–		–	

AOR, adjusted OR; WIC, Women, Infants and Children.

Responsive Style: ^a^Model 1: adjusted by infant age. ^b^Model 2: Model 1 + mother’s age, non-Hispanic White and Education. ^c^Model 5: Model 2 + perception of infant’s weight. Laissez-Faire Style: ^a^Model 1: adjusted by the age of the infant, household income and food security. ^b^Model 2: Model 1 + mother’s age and education. ^c^Model 3: Model 2 + WIC enrolment. ^d^Model 4: Model 3 + Mother’s weight perception. ^e^Model 5: Model 4 + pacifier use and infant’s insurance. Pressuring Style: ^a^Model 1: adjusted by the age of the infant, household income, food security and water security. ^b^Model 2: Model 1 + marital status, non-hispanic white and education. ^c^Model 3: Model 2 + any prenatal care and WIC enrolment. ^d^Model 4: Model 3 + Mother’s risk for anxiety. ^e^Model 5: Model 4 + infant’s insurance and perception of infant’s weight. ^f^Model 6: Model 5 + dietary guidelines. Indulgence Style: ^a^Model 1: adjusted by the age of the infant. ^b^Model 2: Model 1 + mother’s age and marital status. ^c^Model 3: Model 2 + any prenatal care. ^d^Model 4: Model 3 + mother’s risk for anxiety. ^e^Model 5: Model 4 + infant’s insurance. Restrictive style: ^a^Model 1: adjusted by the age of the infant, household income, food security and water security. ^b^Model 2: Model 1 + mother’s age and non-hispanic white. ^c^Model 3: Model 2 + WIC enrolment. ^d^Model 4: Model 3 + mother’s risk for depression. ^e^Model 5: Model 4 + infant’s insurance.

* *P* < 0.05.

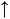
 Increasing the likelihood of exhibiting a feeding style, 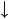 Decreasing the likelihood of exhibiting a feeding style.

## Discussion

Our study identified socio-ecological factors associated with CFS within three socio-ecological levels: household and socio-demographic characteristics, maternal perinatal characteristics and infant and dietary characteristics (Fig. 2). At the household and socio-demographic characteristics level, household characteristics and maternal socio-demographic factors were associated with NRP feeding styles. At the maternal perinatal characteristics level, pregnancy and prenatal care, and maternal mental health factors were associated with NRP feeding styles. Lastly, at the infant and dietary characteristics level, infant characteristics and background factors were associated with NRP feeding styles. Furthermore, no associations were found between CFS and meeting infant dietary guidelines. To our knowledge, this is the first study in Nevada focusing on CFS as a predictor of ECO. This is especially important in the context of urban areas in Nevada because of the high prevalence of ECO. Our study provides insights into socio-ecological factors that cause dissimilarities in CFS that could be potentially used to tailor educational and intervention approaches to address disparities in ECO.

Concerning household characteristics, mothers in lower-income households were more likely to be NRP feeders. This is consistent with previous findings that suggest that mothers living in low-income households are more worried about their infants’ hunger and are less likely to identify hunger and satiety cues^(17)^, thus increasing the likelihood of NRP feeding behaviours. Additionally, if a mother is at risk for water insecurity, it increases the likelihood of being an NRP feeder. Individuals are at risk for water insecurity if they lack water availability, accessibility, use and stability^(40)^. As far as we know, our study is one of the first to study the association between water insecurity and NRP feeding. This is important because, due to climate changes, water availability may be lower at higher costs, thus generating stress on the caregivers’ because they are competing financially with other priorities. This, in turn, may impact a caregiver’s ability to practice RP feeding due to time and opportunity costs associated with water insecurity^(40)^. Water security should continue to be monitored because Nevada is a part of the US Southwest region that is currently going through drought and water shortages^(41)^. This is heightened for Clark County, as it is in the middle of a desert with a limited water supply. Therefore, this finding is important, especially in Clark County, Nevada, and may be useful in identifying ways to support families better in this region.

In our study, young mothers (aged 18–24) were more likely to be NRP feeders than adult mothers. Not many studies have focused on maternal age and feeding styles^(22–25,30–33,36,38)^. However, past research observed that mothers who have gained experience over time were more confident in feeding responsively^(16)^. This suggests that young mothers may have less practice and understanding of RP feeding than adult mothers, so they may be more likely to feed non-responsively. Secondly, consistent with previous research, our results suggest that mothers with higher education are less likely to practice NRP feeding styles. Prior studies have shown that maternal education is strongly associated with adequate eating behaviours and RP feeding styles^(20)^. Mothers with higher incomes and education were found to believe in their infant’s ability to recognise their hunger and satiety cues^(16)^. It is plausible to assume that mothers with higher education levels have more access to knowledge on feeding practices and, therefore, are more aware of their infant’s cues.

Surprisingly, mothers had lower odds of being NRP feeders when they did not receive prenatal care. There is a lack of studies focused on prenatal care and its impact on RP feeding styles; therefore, there are no viable explanations for why we observed this association. However, there may be no difference between the prenatal and non-prenatal groups, as we do not know if RP feeding is even discussed during visits. Other studies on infant feeding explained that prenatal visits tend to emphasise breastfeeding practices, complementary feeding and adequate nutrition but not feeding styles^(42)^. We found that when a mother was not enrolled in WIC, they had a higher probability of being a NRP feeder. Not only is WIC a nutritional supplementation programme that provides nutrition education and food benefits, but it has also been uncovered that WIC staff have the resources to educate mothers on identifying and responding to their infant’s hunger and satiety cues^(29)^. Therefore, mothers enrolled in WIC might obtain more RP feeding advice than mothers who are not, causing non-enrolees to be more likely to be NRP feeders.

We found that a mild risk for depression and a moderate to severe risk for anxiety decreased the likelihood of a mother being an NRP feeder. This is inconsistent with previous findings because they have shown that depression and anxiety increase the probability of an NRP feeding style^(20)^. Skewed results may explain these differences due to the stigma associated with mental health^(20)^. However, the negative emotional response from mental health may cause mothers to reduce their capacity for interaction and engagement to feed responsively and their capacity to feed non-responsively^(16)^.

In relation to infant characteristics, a mother had a higher potential to be an NRP feeder if their infant had non-government insurance. No other studies have investigated the relationship between infant insurance and CFS. However, individuals with non-government insurance (e.g. private insurance) have been shown to have higher access to high-quality care and higher diagnoses for allergies and dietary restrictions^(43,44)^. Therefore, it is likely that increased access to care and testing may also increase caregivers’ knowledge of their infant’s allergies and dietary restrictions, thus causing them to present more NRP feeding styles. Our study found that mothers were more likely to be NRP feeders if they perceived their infant as having normal weight. This is inconsistent with previous studies that suggested mothers who are more worried about their infant’s weight are more likely to be NRP feeders^(14)^. A probable explanation for this finding is that mothers who perceive their children as having normal weight may not care about how they feed their infants, thus causing them not to practice RP feeding styles.

Contrary to what was expected, there were no independent associations between meeting infant dietary guidelines and RP feeding. Corroborating our findings, previous studies investigating this association explained that although caregivers meet their infants dietary guidelines, they may lack the skills to feed responsively^(20)^. Barriers these studies mentioned to RP feeding included balancing milk consumption recommendations and infant feeding cues, recognising and responding to their infant’s cues and a mother’s ability to soothe without food^(20)^. Although there were no significant associations, further studies should be conducted to understand the relationship between RP feeding and infant feeding because prior studies have observed that RP feeding helps infants develop healthy dietary habits and learn to self-regulate^(28)^.

Our study has strengths and limitations to consider when interpreting our findings. This study was cross-sectional; therefore, we cannot infer causation. Despite this, a strength is that our study provides a baseline of specific factors that are associated with RP and NRP feeding styles, which future researchers can use to create hypotheses for further studies. Second, this study utilised self-reported measures, such as maternal mental health and caregivers’ feeding practices and beliefs, causing self-reported bias. However, a strength is that the questions used for these measures are from valid and reliable instruments. Third, this study utilised a snowball convenience sample of mothers and caregivers with infants under two years old across Clark County, Nevada. While most of the sample was recruited through paid social media advertisements, to ensure diverse socio-demographic representation the survey was advertised at birth, paediatric offices, paediatric dentist and lactation centres within Clark County. As a result, our convenience sample has similar demographic characteristics (e.g. household income, marital status, ethnicity and education) to Clark County’s available data^(45)^. Fourth, this study is limited to the mothers and caregivers of one large urban geographical area in the USA. However, since Clark County is the largest urban area in the state of Nevada and with a very diverse socio-demographic population, findings may be generalised to similar urban areas in high-income countries.

The use of the HWIAS survey can be considered a limitation due to not being validated in the USA or other high-income countries; however, neither is any other water insecurity survey^(46)^. Regardless, water security has become a global concern and it has been becoming more prevalent in the USA^(40,41,46,47)^. Therefore, measuring water insecurity, even with a question that is not validated, is essential to uncover problems that would otherwise stay invisible. For example, water insecurity increases an individual’s expenses on water bottles and treatment devices, as they lack trust in tap water quality^(47)^, which can affect the economic stability of the family, that in turn, corroborating our findings can lower the odds of RP feeding. Sixth, there may be some temporality issues with some of the survey questions (e.g. food and water insecurity) due to measuring risk in the last 12 months. Lastly, we opted to classify our RP and NRP outcomes as binary rather than continuous variables after conducting sensitivity analysis and finding similar results (data not shown). Our option to use binary variables relies on our hypothesis to identify the association between socio-ecological factors and each CFS. We acknowledge that this will reduce the variation of the outcomes and is less informative than using continuous variables, but the goal of the study was to not understand the different dimensions of the feeding styles, but to understand the predominant feeding styles of an individual (knowing other feeding styles play a role) and what factors are causing those feeding styles. We are less interested in how much of that feeding style they possess over another. Additionally, the way the survey was developed, the feeding styles were not mutually exclusive, which brought the limitation of a measurement issue. Although limited to countries other than the USA, a few similar studies have looked at the factors influencing CFS^(14,16–20)^.

Our study identified socio-ecological factors associated with dissimilarities in CFS in Nevada that can contribute to disparities in ECO development, which is a public health crisis. This study is innovative because it identified factors that other studies have not. For example, associations between maternal age, infant insurance, water insecurity, prenatal care, WIC enrolment and a caregivers’ feeding style. Prior studies have found that RP and NRP feeding styles influence ECO, with RP feeding nurturing healthy eating and growth and NRP feeding creating overnutrition and obesity^(14)^. Therefore, longitudinal studies investigating the mechanisms through which RP feeding can improve ECO should be conducted. These longitudinal studies should consider clarifying the role of cofounders influencing RP feeding found in our study, such as water insecurity, anxiety and depression, and looking at current prenatal care counselling on RP care. There is an opportunity for policies and interventions to include in their programmatic activities informational or educational resources to support RP feeding. In addition, further qualitative investigation should explore how caregivers could overcome barriers to RP feeding skills, which would provide new insight into prevention mechanisms for ECO and could inform guidelines for educating caregivers about infant feeding styles and behaviours as a way of ECO prevention.

## Conclusion

Socio-ecological factors, including household, maternal socio-demographic, infant characteristics, pregnancy and prenatal care and maternal mental health, were associated with caregivers’ NRP feeding styles in a diverse sample of caregiver-infant dyads living in urban areas in Nevada. These findings can be used to inform educational approaches to support RP feeding to prevent ECO, a public health crisis in the USA.

## Financial support

Amanda Castelo Saragosa received a scholarship from the Health Resources and Services Administration (HRSA) Award Number 1 T52HP46756-01-00 awarded to the School of Public Health, University of Nevada, Las Vegas (UNLV), to help fund this study.

## Conflict of interest

There are no conflicts of interest.

## Authorship

A.C.S. and G.B. developed the concept and design of the study. A.C.S. collected and managed the data and conducted the formal analysis supervised by G.B. and S.M. S.M., C.J. and A.C. contributed to the conceptualisation of the study. A.C.S. wrote the original draft of the manuscript. G.B., S.M., C.J. and A.C. critically revised and edited the manuscript. All authors approved the final version of the manuscript.

## Ethics of human subject participation

This study was conducted according to the guidelines laid down in the Declaration of Helsinki, and all procedures involving research study participants were approved by the University of Nevada, Las Vegas’s Institutional Review Board (Protocol UNLV-2022-372). Written informed consent was obtained from all subjects.

## Supplementary material

For supplementary material accompanying this paper visit https://doi.org/10.1017/S1368980025000096

## Supporting information

Castelo Saragosa et al. supplementary material Castelo Saragosa et al. supplementary material
