# Supplementary material for: Factors associated with caregiver responsive and non-responsive feeding styles in Clark County, Nevada
Source: Public Health Nutr. 2025 Jan 30;28(1):e42. doi: 10.1017/S1368980025000096 (PMC11883572; doi:10.1017/S1368980025000096)
Supplement: Castelo Saragosa et al. supplementary material [file S1368980025000096sup001.docx]

# **Appendix A: STROBE Checklist**

STROBE Statement—Checklist of items that should be included in reports of ***cross-sectional studies***

|  | **Item No** | **Recommendation** | **Page No** |
| --- | --- | --- | --- |
| **Title and abstract** | 1 | (*a*) Indicate the study’s design with a commonly used term in the title or the abstract | 1 |
|  |  | (*b*) Provide in the abstract an informative and balanced summary of what was done and what was found | 1-2 |
| **Introduction** | | |  |
| Background/rationale | 2 | Explain the scientific background and rationale for the investigation being reported | 3-4 |
| Objectives | 3 | State specific objectives, including any prespecified hypotheses | 4 |
| **Methods** | | |  |
| Study design | 4 | Present key elements of study design early in the paper | 4 |
| Setting | 5 | Describe the setting, locations, and relevant dates, including periods of recruitment, exposure, follow-up, and data collection | 5 |
| Participants | 6 | (*a*) Give the eligibility criteria, and the sources and methods of selection of participants | 5 |
| Variables | 7 | Clearly define all outcomes, exposures, predictors, potential confounders, and effect modifiers. Give diagnostic criteria, if applicable | 6-10 |
| Data sources/ measurement | 8* | For each variable of interest, give sources of data and details of methods of assessment (measurement). Describe comparability of assessment methods if there is more than one group | 5-10 |
| Bias | 9 | Describe any efforts to address potential sources of bias | 16-17 |
| Study size | 10 | Explain how the study size was arrived at | 5 |
| Quantitative variables | 11 | Explain how quantitative variables were handled in the analyses. If applicable, describe which groupings were chosen and why | 6-10 |
| Statistical methods | 12 | (*a*) Describe all statistical methods, including those used to control for confounding | 10-11 |
|  |  | (*b*) Describe any methods used to examine subgroups and interactions | 10-11 |
|  |  | (*c*) Explain how missing data were addressed | n/a |
|  |  | (*d*) If applicable, describe analytical methods taking account of sampling strategy | n/a |
|  |  | (*e*) Describe any sensitivity analyses | 10-11 |
| **Results** | | |  |
| Participants | 13* | (a) Report numbers of individuals at each stage of study—eg numbers potentially eligible, examined for eligibility, confirmed eligible, included in the study, completing follow-up, and analysed | 11-12 |
|  |  | (b) Give reasons for non-participation at each stage | n/a |
|  |  | (c) Consider use of a flow diagram | n/a |
| Descriptive data | 14* | (a) Give characteristics of study participants (eg demographic, clinical, social) and information on exposures and potential confounders | 11-12 |
|  |  | (b) Indicate number of participants with missing data for each variable of interest | n/a |
| Outcome data | 15* | Report numbers of outcome events or summary measures | 11-12 |
| Main results | 16 | (*a*) Give unadjusted estimates and, if applicable, confounder-adjusted estimates and their precision (eg, 95% confidence interval). Make clear which confounders were adjusted for and why they were included | 12-13 |
|  |  | (*b*) Report category boundaries when continuous variables were categorized | 12-13 |
|  |  | (*c*) If relevant, consider translating estimates of relative risk into absolute risk for a meaningful time period | n/a |
| Other analyses | 17 | Report other analyses done—eg analyses of subgroups and interactions, and sensitivity analyses | n/a |
| **Discussion** | | |  |
| Key results | 18 | Summarise key results with reference to study objectives | 13-14 |
| Limitations | 19 | Discuss limitations of the study, taking into account sources of potential bias or imprecision. Discuss both direction and magnitude of any potential bias | 16-17 |
| Interpretation | 20 | Give a cautious overall interpretation of results considering objectives, limitations, multiplicity of analyses, results from similar studies, and other relevant evidence | 14-16 |
| Generalisability | 21 | Discuss the generalisability (external validity) of the study results | 16-18 |
| **Other information** | | |  |
| Funding | 22 | Give the source of funding and the role of the funders for the present study and, if applicable, for the original study on which the present article is based | n/a |

*Give information separately for exposed and unexposed groups.

**Note:** An Explanation and Elaboration article discusses each checklist item and gives methodological background and published examples of transparent reporting. The STROBE checklist is best used in conjunction with this article (freely available on the Web sites of PLoS Medicine at http://www.plosmedicine.org/, Annals of Internal Medicine at http://www.annals.org/, and Epidemiology at http://www.epidem.com/). Information on the STROBE Initiative is available at www.strobe-statement.org.

# **Appendix B: Infant Feeding Style Questionnaire**

(Thompson et al., 2009)

| **Laissez-faire feeding style questions, 2023.** | |
| --- | --- |
| **LF1** | When (name of child) has/had a bottle, I prop/propped it up |
| **LF2** | (Child) watches TV while eating |
| **LF3** | I watch TV while feeding (child) |
| **LF4** | I think it is okay to prop an infant’s bottle |
| **LF5** | It’s okay for a toddler to walk around while eating as long as s/he eats |
| **LF6** | I keep track of what food (child) eats |
| **LF7** | I keep track of how much food (child) eats |
| **LF8** | I make sure (child) does not eat sugary food like candy, ice cream, cakes, or cookies |
| **LF9** | I make sure (child) does not eat junk food like potato chips, Doritos, and cheese puffs |
| **LF10** | A toddler should be able to eat whatever s/he wants for snacks |
| **LF11** | A toddler should be able to eat whatever s/he wants when eating out at a restaurant |

| **Pressuring feeding style questions, 2023.** | |
| --- | --- |
| **PR1** | Try to get (child) to finish his/her food |
| **PR2** | If (child) seems full, encourage to finish anyway |
| **PR3** | Try to get (child) to finish breastmilk or formula |
| **PR4** | Try to get (child) to eat even if not hungry |
| **PR5** | Insist re-try new food refused at same meal |
| **PR6** | Praise after each bite to encourage finish food |
| **PR7** | Important for toddler finish all food on his/her plate |
| **PR8** | Important for infant finish all milk in his/her bottle |
| **PR11** | Give/gave (child) cereal in the bottle |
| **PR12** | Cereal in bottle helps infant sleep thru the night |
| **PR13** | Putting cereal in bottle good b/c helps infant feel full |
| **PR14** | An infant <6 months needs more than formula or breastmilk to be full |
| **PR15** | An infant <6 months needs more than formula or breastmilk to sleep through the night |
| **PR16** | When (child) cries, I immediately feed him/her |
| **PR17** | Best way to make infant stop crying is to feed |
| **PR18** | Best way to make toddler stop crying is to feed |
| **PR19** | When infant cries, usually means s/he needs to be fed |

| **Restrictive feeding style questions, 2023.** | |
| --- | --- |
| **RS1** | I carefully control how much (child) eats |
| **RS2** | I am very careful not to feed (child) too much |
| **RS3** | Important parent has rules re: how much toddler eats |
| **RS4** | Important parent decides how much infant should eat |
| **RS5** | I let (child) eat fast food |
| **RS6** | I let (child) eat junk food |
| **RS7** | A toddler should never eat fast food |
| **RS8** | An infant should never eat fast food |
| **RS9** | A toddler should never eat sugary food like cookies |
| **RS10** | A toddler should never eat junk food like chips |
| **RS11** | A toddler should only eat healthy food |

| **Responsive feeding style questions, 2023.** | |
| --- | --- |
| **RP1** | (Child) lets me know when s/he is full |
| **RP2** | (Child) lets me knows when s/he is hungry |
| **RP3** | I let (child) decide how much to eat |
| **RP4** | I pay attention when (child) seems to be telling me that s/he is full or hungry |
| **RP5** | I allow (child) to eat when s/he is hungry |
| **RP6** | Child knows when s/he is full |
| **RP7** | Child knows when hungry, needs to eat |
| **RP8** | Talk to (child) to encourage to drink formula/breastmilk |
| **RP9** | Talk to (child) to encourage him/her to eat |
| **RP10** | Show (child) how to eat by taking a bite or pretending |
| **RP11** | I will retry new foods if they are rejected at first |
| **RP12** | Important to help or encourage a toddler to eat |

| **Indulgence feeding style questions, 2023.** | |
| --- | --- |
| **ID1** | Allow child watch TV while eating if s/he wants |
| **ID2** | Allow child to eat fast food if s/he wants |
| **ID3** | Allow child to drink sugary drinks/soda if s/he wants |
| **ID4** | Allow child to eat desserts/sweets if s/he wants |
| **ID5** | Toddlers should be allowed to watch TV while eating if they want |
| **ID6** | Toddlers should be allowed to eat fast food if they want |
| **ID7** | Toddlers should be allowed to drink sugary drinks/soda if they want |
| **ID8** | Toddlers should be allowed to eat desserts/sweets if they |
| **ID9** | Allow child watch TV while eating to make sure s/he gets enough |
| **ID10** | Allow child to eat fast food to make sure s/he gets enough |
| **ID11** | Allow child to drink sugary drinks/soda to make sure s/he gets enough |
| **ID12** | Allow child to eat desserts/sweets to make sure s/he gets enough |
| **ID13** | Toddlers should be allowed to watch TV while eating to make sure they get enough |
| **ID14** | Toddlers should be allowed to eat fast food to make sure they get enough |
| **ID15** | Toddlers should be allowed to drink sugary drinks/soda to make sure they get enough |
| **ID16** | Toddlers should be allowed to eat desserts/sweets to make sure they get enough |
| **ID17** | Allow child watch tv while eating to keep him/her from crying |
| **ID18** | Allow child to eat fast food to keep him/her from crying |
| **ID19** | Allow child to drink sugary drinks/soda to keep him/her from crying |
| **ID20** | Allow child to eat desserts/sweets to keep him/her from crying |
| **ID21** | Toddlers should be allowed to watch tv while eating to keep them from crying |
| **ID22** | Toddlers should be allowed to eat fast food to keep them from crying |
| **ID23** | Toddlers should be allowed to drink sugary drinks/soda to keep them from crying |
| **ID24** | Toddlers should be allowed to eat desserts/sweets to keep them from crying |
| **ID25** | Allow child watch tv while eating to keep him/her happy |
| **ID26** | Allow child to eat fast food to keep him/her happy |
| **ID27** | Allow child to drink sugary drinks/soda to keep him/her happy |
| **ID28** | Allow child to eat desserts/sweets to keep him/her happy |
| **ID29** | Toddlers should be allowed to watch tv while eating to keep them happy |
| **ID30** | Toddlers should be allowed to eat fast food to keep them happy |
| **ID31** | Toddlers should be allowed to drink sugary drinks/soda to keep them happy |
| **ID32** | Toddlers should be allowed to eat desserts/sweets to keep them happy |

#

# **Appendix C: Outcome Table**

| **Definitions, classifications, mean, and standard deviation of study outcomes, 2023.** | | | | | |
| --- | --- | --- | --- | --- | --- |
| **# of Items** | **Outcome** | **Definition** | **Classification** | **Mean** | **Standard Deviation** |
| 12 | Responsive Feeding | Parent is attentive to child hunger and satiety cues and monitors the quality of the child’s diet (Thompson et al., 2009) | Responsive  Non-Responsive | 4.06 | 0.03 |
| 11 | Laissez-faire feeding (Non-Responsive) | Parent does not limit infant's diet quality or quantity and shows little interaction with the infant during feeding (Thompson et al., 2009) | Laissez-faire  Not laissez-faire | 2.95 | 0.02 |
| 17 | Pressuring feeding (Non-Responsive) | The parent is concerned with increasing the amount of food the infant consumes and uses food to soothe the infant (Thompson et al., 2009) | Pressuring  Not pressuring | 2.01 | 0.03 |
| 32 | Indulgent feeding (Non-Responsive) | Parent does not set limits on the quantity or quality of food consumed  (Thompson et al., 2009) | Indulgent  Not Indulgent | 1.50 | 0.03 |
| 11 | Restrictive feeding (Non-Responsive) | Parent limits the infant to healthful foods and limits the quantity of food consumed (Thompson et al., 2009) | Restrictive  Not Restrictive | 2.62 | 0.03 |

# **Appendix D: Covariables**

**Definitions, instruments used, questions, scoring, and classifications of study covariables and outcomes in the survey, 2023.**

| **Covariable** | **Definition** | **# of Items** | **Instrument** | **Question(s)** | **Scoring** | **Classification** |
| --- | --- | --- | --- | --- | --- | --- |
| **Household and Sociodemographic Characteristics** | | | | | | |
| Block 1: Household Characteristics | | | | | | |
| Household Income | Measure of the combined incomes of all individuals in a household. | 1 | n/a | Which household income best describes your family's annual household income from  wages, salaries, and business? | n/a | -Low income (less than $49,999)  -Middle income ($50,000-$149,999)  -Upper income (more than $150,000) |
| Food Security | Lack of consistent access to enough food for an active, healthy life (Taher et al., 2022). | 2 | Hunger Vital Sign | (1) Within the past 12 months, we worried whether our food would run out before we got money to buy more.  (2) Within the past 12 months, we bought food that just didn’t last and we didn’t have money to get more. | Never true/ Sometimes true/ Often true.  If an individual answers sometimes true or often true to any of these questions, they are at risk for food insecurity. | -Food secure  -Food insecure |
| Water Security | Lack of consistent access to enough water for productivity and survival (Miller et al., 2021). | 1 | Household Water Insecurity Access Scale | Within the past 12 months, we worried about not having enough money to afford access to clean water (i.e., drinking water, bathing/washing hands, washing clothes, or any other needs). | Never true/ Sometimes true/ Often true.  If an individual answers sometimes true or often true to this question, they are at risk for water insecurity. | -Water Secure  -Water Insecure |
| Block 2: Maternal Sociodemographics | | | | | | |
| Age | Age of the mother. | 1 | n/a | How old is the mother of this child? | n/a | -18 - 24  -25 - 34  -35 – 44 |
| Marital Status | The state of being married or not married. | 1 | n/a | What is your marital status? | n/a | -Living without a partner (single, widowed, separated)  -Living with a partner (married, living together) |
| Non-Hispanic White | Race refers to a person's physical characteristics, like skin color, while ethnicity refers to cultural characteristics like religion, history, language, and customs. (Blakemore, 2021) | 1 | n/a | What is the mother's race?  Is the mother of Latina/Hispanic, or Spanish origin? | Participants were asked to select all race categories that applied.  Participants were then asked to select if the mother was Hispanic.  The two questions were combined and separated into non-Hispanic white and Hispanic white. | -Yes  -No |
| Education | The level of education the mother has obtained. | 1 | n/a | What is the highest level of education of the mother? | n/a | -Secondary or college  -Graduate |
| **Maternal Perinatal Characteristics** | | | | | | |
| Block 3: Pregnancy and Prenatal Care | | | | | | |
| Any prenatal care | If the mother saw a health care professional during pregnancy for prenatal care. | 1 | n/a | Did you visit a primary care doctor or OB/GYN for prenatal care? | n/a | -Yes  -No |
| WIC  Enrollment | Is the participant currently enrolled in WIC and receiving WIC benefits | 1 | n/a | Are you enrolled in your WIC program? | n/a | -Yes  -No |
| Block 4: Maternal Mental Health | | | | | | |
| Depression | An illness that negatively affects how you feel, the way you think, and how you act (Depression, n.d.) | 10 | Edinburgh Postnatal Depression Scale | (1) I have been able to laugh and see the funny side of things.  (2) I have looked forward with enjoyment to things.  (3) I have blamed myself unnecessarily when things went wrong.  (4) I have been anxious or worried for no good reason.  (5) I have felt scared or panicky for no good reason.  (6) Things have been getting to me.  (7) I have been so unhappy that I have had difficulty sleeping.  (8) I have felt sad or miserable.  (9) I have been so unhappy that I have been crying.  (10) The thought of harming myself has occurred to me. | Scale from 0-3.  Scores:  -No or minimal depression risk (0-6)  -Mild depression risk (7-13)  -Moderate depression risk (14-19)  -Severe depression risk (19-30) | -None or minimal depression risk  -Mild depression risk  -Moderate/Severe Depression Risk |
| Anxiety | An emotion characterized by feelings of tension, fear, dread, uneasiness, worried thoughts, and physical changes (like increased blood pressure). (American Psychological Association, n.d.) | 7 | General Anxiety Disorder- 7 | (1) Feeling nervous, anxious or on edge.  (2) Not being able to stop or control worrying.  (3) Worrying too much about different things.  (4) Trouble relaxing.  (5) Being so restless that it is hard to sit still.  (6) Becoming easily annoyed or irritable.  (7) Feeling afraid, as if something awful might happen. | Scale from 0-3.  Scores:  -Minimal anxiety risk (0-4)  -Mild anxiety risk (5-9)  -Moderate anxiety risk (10-14)  -Severe anxiety risk (15-21). | -Minimal anxiety risk  -Mild anxiety risk  -Moderate to Severe anxiety risk |
| Parental  Burnout | An individual’s emotional distress, exhaustion, and feelings from being a parent (Aunola et al., 2021). | 5 | Brief Parental Burnout Scale | (1) I’m so tired out by my role as a parent that sleeping doesn’t seem like enough.  (2) I have the sense that I’m really worn out as a parent.  (3) I have the impression that I’m looking after my child(ren) on autopilot (I do what I’m supposed to do for my child(ren), but nothing more).  (4) I am no longer able to show my child(ren) how much I love them.  (5) I feel like I can’t take any more as a parent. | A “daily,” B “Once or twice a week,” and C “More seldom/never.”  If a parent answers “A” to at least one question or “B” to at least two questions, they are at risk for parental burnout. | -Burnout Risk  -No Burnout Risk |
| Maternal Weight Perception | Personal evaluation of one’s weight. | 1 | n/a | How do you describe your weight? | n/a | -Underweight  -Normal weight  -Overweight |
| **Infant and Dietary Characteristics** | | | | | | |
| Block 5: Infant Characteristics and Background | | | | | | |
| Pacifier use | The infant is using pacifiers | 1 | n/a | In the last 24 hours, has your baby used a pacifier? | n/a | -Yes  -No |
| Covered by health insurance | The type of insurance the infant is covered by. | 1 | n/a | What type of medical insurance does your child have | n/a | -Government  -Non-Government |
| Perception of Child’s Weight | Caregivers' perception of infants' weight. | 1 | n/a | How would you describe your child’s weight? | n/a | -Underweight  -Normal weight  -Overweight |
| Block 6: Infant Dietary Guidelines | | | | | | |
| Infant Dietary Guidelines | **Exclusive breastfeeding <6 months:**  Exclusive breastfeeding means feeding your baby only breast milk, not any other foods or liquids (including infant formula or water), except for medications or vitamin and mineral supplements. (CDC)  **Minimum Dietary Diversity > 7 months:**  Complementary foods are foods or drinks other than breast milk or infant formula (e.g., infant cereals, fruits, vegetables, water). (CDC) | 1 |  | From Yesterday morning until this morning, what has your child eaten?((1) breast milk, (2) grains/roots/tubers/plantains, (3) pulses/nuts/seeds, (4) dairy products, (5) flesh foods, (6) eggs, (7) vitamin A rich fruits/vegetables, and (8) other fruits/vegetables) | If the child was <6 months and was fed breastmilk only, they were classified as exclusive breastfeeding.  If the child was >7 months and was fed at least five out of the eight food groups, they were classified as having minimum dietary diversity.  They were classified as meeting dietary guidelines if their child was exclusively breastfed <6 months or had minimum dietary diversity >7 months.  They were classified as not meeting guidelines if their child was not exclusively breastfeed <6 months or if they did not have minimum dietary diversity >7 months. | -Met guidelines  -Did not meet guidelines |
| **Outcome: Caregivers’ Feeding Styles** | | | | | | |
| Caregiver Feeding Styles | How caregivers maintain or modify their child's eating behaviors and feeding environment(2,4,19 | 83 | Infant Feeding Style Questionnaire | Appendix B | Scale 1-5.  Scoring:  -Take the overall mean score of the feeding style questions.  -If an individual scores above that mean, they are classified as having a feeding style.  -If an individual scores below that mean, they are classified as not having that feeding style. | Appendix C |
